# Mechanisms Linking Vitamin D Deficiency to Impaired Metabolism: An Overview

**DOI:** 10.1155/2022/6453882

**Published:** 2022-07-06

**Authors:** Nurulmuna Mohd Ghozali, Nelli Giribabu, Naguib Salleh

**Affiliations:** Department of Physiology, Faculty of Medicine, University of Malaya, Lembah Pantai, Kuala Lumpur 59100, Malaysia

## Abstract

Vitamin D deficiency is a common health problem worldwide. Despite its known skeletal effects, studies have begun to explore its extra-skeletal effects, that is, in preventing metabolic diseases such as obesity, hyperlipidemia, and diabetes mellitus. The mechanisms by which vitamin D deficiency led to these unfavorable metabolic consequences have been explored. Current evidence indicates that the deficiency of vitamin D could impair the pancreatic *β*-cell functions, thus compromising its insulin secretion. Besides, vitamin D deficiency could also exacerbate inflammation, oxidative stress, and apoptosis in the pancreas and many organs, which leads to insulin resistance. Together, these will contribute to impairment in glucose homeostasis. This review summarizes the reported metabolic effects of vitamin D, in order to identify its potential use to prevent and overcome metabolic diseases.

## 1. Introduction

Vitamin D (calciferol) refers to a group of fat-soluble secosteroids that exists in two forms: vitamin D_2_ (ergocalciferol) and vitamin D_3_ (cholecalciferol). Vitamin D_2_ is derived from plant's ergosterol upon exposure to ultraviolet B (UVB) light [1], whereas vitamin D_3_ is derived from 7-dehydrocholesterol (7DHC) found in the human and animal skin following exposure also to UVB light [2]. The main exogenous sources of vitamin D_2_ are plants, plankton, and fungi [3], whereas the main sources of vitamin D_3_ are dairy products, fish, meat, and poultry [4, 5]. Additionally, both types of vitamin D are currently available as dietary supplements and in the form of fortified foods [6]. In the body, vitamin D_2_ and vitamin D_3_ will undergo hydroxylation in the liver involving the 25-hydroxylase (CYP2R1) enzyme to form ercalcidiol (25(OH)D_2_) and calcidiol (25(OH)D) [6, 7] where these will then bind to vitamin D binding protein (DBP) in the circulation [8]. Calcidiol is the major circulating form of vitamin D [9], and therefore, its serum concentration can be used to measure the vitamin D status [10]. In the kidney, calcidiol undergoes another hydroxylation process mediated by the 1*α*-hydroxylase (CYP27B1) enzyme to form calcitriol (1,25(OH)_2_), which is a fully active hormone that is responsible for most (if not all) of the vitamin D biological actions [6, 7]. At tissue levels, vitamin D in its active form (calcitriol) binds to the vitamin D receptor (VDR), which is an intracellular protein, and the vitamin D-VDR complex translocates into the nucleus to bind to target genes and modify the gene expression [11, 12]. Additionally, vitamin D could also induce VDR expression and activation by auto-regulating the expression of the *Vdr* gene and inducing the transcription of *Vdr* gene itself [11, 13]. Classically, vitamin D is known for its role in maintaining bone health by regulating calcium homeostasis and by exerting direct action on the cartilage and bones to promote normal skeletal development [7]. However, in the past few decades, researchers have begun to explore the extra-skeletal effects of vitamin D [14–16] including metabolism, in view that VDR and the enzymes that are linked to vitamin D have been found in many tissues of the body [17].

### 1.1. Vitamin D and Metabolisms

There is growing evidence that vitamin D has a significant role in metabolism, in particular in regulating the glucose homeostasis [18–21]. Vitamin D deficiency has been identified as a global health issue by epidemiological studies [22, 23] and is defined by serum calcidiol levels of lower than 50 nmol/L or 20 ng/ml [24]. In addition to higher risk of development of rickets and osteomalacia in the growing skeleton [7], osteoporosis in the aging skeleton [25], and stress fractures in the physically active individuals [26], recent studies found that vitamin D deficiency could cause impairment of insulin secretion and trigger development of insulin resistance [27, 28]. In fact, insufficient blood levels of vitamin D have been associated with impaired glucose tolerance and hyperinsulinemia [29, 30], often caused by obesity, and these pose major risk for diabetes mellitus (DM) development [31]. *Vice versa*, studies have also found that high incidence of DM was observed in individuals who are deficient in vitamin D [32–35].

Apart from impaired glucose metabolism, vitamin D deficiency has also been associated with obesity [36] and the deficiency of vitamin D was found to be more prevalent in obese individuals [37, 38]. Furthermore, lower serum vitamin D levels were observed in a person with higher body mass index (BMI) [39] and weight loss was found to lead to higher serum vitamin D levels [40]. Alternatively, vitamin D supplementation would significantly decrease the body weight, BMI, as well as waist and hip circumferences [40, 41]. It is unclear what contributes towards the inverse relationship between serum vitamin D levels and BMI; however, it is likely that the larger volume of body fluid in overweight and obese subjects would result in lower serum concentrations of vitamin D [42]. Other mechanisms, however, have not been extensively explored. In addition to causing impaired glucose metabolism, deficiency in vitamin D could also cause dyslipidemia, a condition frequently linked to impaired glucose homeostasis [43]. Vitamin D supplementation was found to lower the blood total cholesterol, low-density lipoproteins (LDL), and triglycerides (TG) levels [44]. Besides, a study has found that LDL and TG levels were inversely correlated with serum vitamin D levels [43] and low high-density lipoproteins (HDL) levels were associated with low serum vitamin D levels [43, 45]. In view of these, vitamin D has been proposed to be clinically useful as an adjuvant therapy for statin in the treatment of hypercholesterolemia [46, 47]. Although the exact mechanisms are unclear, it was suggested that vitamin D could affect cholesterol metabolism by either decreasing the cholesterol absorption or reducing the endogenous cholesterol production [47]; however, these have yet to be proven.

In view of the evidence pointing towards the metabolic effect of vitamin D, this review aims at summarizing the current understanding on the role of vitamin D in body metabolism and how its deficiency could impair the glucose homeostasis that would lead to insulin resistance. Several evidences have shown the association between serum vitamin D levels and insulin resistance, which pointed towards the possible protective effects of vitamin D on pancreatic *β*-cell function [48]. Additionally, vitamin D could also help to enhance insulin sensitivity [49] and ameliorate chronic inflammation [50], oxidative stress [51], and apoptosis [52] in the pancreas and other insulin-responsive tissues [20, 30].

In order to gain understanding on the mechanisms linking vitamin D deficiency to impaired metabolism, the PubMed database (https://www.ncbi.nlm.nih.gov/pubmed) was used to search for articles published between 1995 and 2021 based on the following keywords or their combinations: vitamin D; vitamin D deficiency; insulin resistance; obesity; diabetes; glucose; metabolism; insulin secretion; insulin sensitivity; inflammation; oxidative stress; or apoptosis. In this narrative review, the relevant articles and other related reference lists were evaluated. The findings from animal experiments, human clinical trials, and *in vitro* studies are conveyed, whereas the molecular mechanisms proposed by the researchers were evaluated and discussed accordingly.

## 2. Studies Linking Vitamin D Deficiency to Impaired Metabolism

### 2.1. Animal Studies

Lower serum glucose levels, improved activities of enzymes related to glucose metabolic pathways, restoration of glucose homeostasis, and reduced pancreatic and liver damage were observed following intraperitoneal injections of vitamin D (7 ng/gm) daily to alloxan-induced diabetic female albino mice for fifteen (15) consecutive days [53]. In addition, a study has also found enhanced glucose uptake, improved glucose metabolism, and reduced body oxidative stress level following oral supplementation of 67 IU/kg/day cholecalciferol for 8 weeks to diet-induced vitamin D-deficient obese C57BL/6J male mice [54]. A study also indicated reduced body weight as well as reduced blood glucose level, and complete restoration of insulin sensitivity following the treatment of diet-induced obese, insulin-resistant C57BL/6J male mice with 3000 IU/kg/day with vitamin D for seven (7) consecutive days [55]. In streptozotocin (STZ)-induced diabetic male Sprague-Dawley (SD) rats, intraperitoneal injections of 20,000 IU/kg cholecalciferol resulted in a significant decrease in the fasting plasma glucose (FPG), HbA1c, and an improvement in insulin and IGF-1 levels [56]. In the meantime, intraperitoneal injection of 7 *μ*g/kg vitamin D at a frequency of three (3) times a week for eight (8) weeks to high-fat, high-sugar (HFHS) diet-induced obese C57BL/6J male mice resulted in reduced body weight, improvement in overall systemic glucose tolerance, restoration of insulin signaling, and reverted hepatic myosteatosis [57].

In another study, enhanced insulin resistance, which was evidenced by elevated homeostatic model assessment for insulin resistance (HOMA-IR) index, was observed when STZ-induced male SD rats were fed with vitamin D-deficient diet, and this was later reversed by the consumption of normal diet containing vitamin D [58]. In the meantime, 9–12-week treatment of 20 *μ*g/kg calcipotriol to diet-induced obese C57BL/6J wild-type (WT) mice was associated with suppressed liver inflammation and hepatic steatosis as well as improvement in the overall insulin sensitivity [59]. In contrast to the above studies, a study by Liu et al. showed that no direct association was observed between vitamin D deficiency and obesity, insulin resistance, and hepatic lipid accumulation in HFD-induced vitamin D-deficient young ICR male mice [60]. Furthermore, they proposed that vitamin D deficiency could lead to enhance fatty acid *β*-oxidation and increases the adipose tissue energy expenditure, which might result in the overall reduction in body weight, increased plasma insulin levels, and increased hepatic lipid accumulation [60].

### 2.2. Human Studies

Vitamin D supplementation was reported to help to improve the metabolic parameters associated with insulin resistance and DM in human subjects [61]. In a double-blinded, randomized, placebo-controlled trial in obese subjects, weekly supplementation with 25,000 IU cholecalciferol orally for 3 months together with hypocaloric diet resulted in improved insulin sensitivity [62]. In the meantime, another double-blind, randomized clinical study on prediabetic, vitamin D-deficient human subjects revealed significant improvement in insulin sensitivity and reduced progression toward overt DM following six (6)-month treatment with oral vitamin D_3_ (50,000 IU), weekly for three (3) months, followed by once-a-month treatment for the next three (3) months [63]. In a randomized controlled trial, daily supplementation with 2000 IU cholecalciferol orally for 3 months to human subjects with type 2 DM (T2DM) with vitamin D deficiency has resulted in decreased HOMA-IR index as well as HbA1c levels, a marked increase in HDL level, a decreased in TG/HDL ratio, and a reduced level of endogenous and oxidative DNA damages in the lymphocytes [64]. Additionally, a double-blind randomized clinical trial revealed that vitamin D-deficient obese individuals with T2DM who received weekly treatment with 50,000 IU oral vitamin D for eight (8) weeks had a significant decrease in HbA1c levels; however, no significant changes in FPG, insulin, HOMA-IR index, and quantitative insulin sensitivity check index (QUICKI) were observed [65].

A double-blind, placebo-controlled, randomized clinical trial involving human subjects with serum 25(OH)D level ≤50 nmol/L and a BMI of 30–40 kg/m^2^ showed that weekly supplementation with 50,000 IU vitamin D_3_ for twelve (12) weeks resulted in a significant decrease in the BMI and fasting blood glucose (FBG) levels [66]. The benefits of vitamin D in improving the metabolic parameters were also seen in post-menopausal women, where a significant reduction in the metabolic syndrome risk profile including hyperglycemia, hypertriglyceridemia, and HOMA-IR was observed following daily supplementation with oral vitamin D_3_ (1000 IU) for nine (9) months [67]. Furthermore, premenopausal women with vitamin D insufficiency receiving 20,000 IU oral cholecalciferol weekly for twenty-four (24) weeks had a significant improvement in insulin resistance indices including HOMA-IR and QUICKI, despite no significant changes in the area under the curve (AUCgluc) for glucose tolerance [68].

In contrast to the benefits of vitamin D in improving the metabolic profiles in obese and post-menopausal subjects, a single-blind randomized control trial in diabetic pregnant women with vitamin D deficiency, however, reported no improvement in insulin resistance or glycemic control following 60,000 IU of oral vitamin D_3_ supplementation once a month until delivery [69]. Similarly, a randomized, placebo-controlled, double-blind trial on women suffering from polycystic ovarian syndrome (PCOS) with vitamin D deficiency demonstrated no significant changes in the fasting serum insulin and FBG levels and the HOMA-IR index after supplementation with 50,000 IU oral vitamin D_3_ once every 20 days for two (2) months [70].  Table 1 summarizes the reported effects of vitamin D on metabolism in animals, while Table 2 presents a list of interventional clinical trials of the benefits of vitamin D supplementation in improving metabolic profiles in humans.

## 3. Mechanisms Underlying the Action of Vitamin D in Improving Impaired Metabolism

### 3.1. Vitamin D Improves Pancreatic *β*-cell Functions

Functional, pancreatic *β*-cells play important role in maintaining the blood glucose homeostasis [71, 72]. These cells adapt to an excessive blood glucose level by increasing the insulin secretion, and the latter is further exaggerated in the state of insulin resistance [73, 74]. Compensatory hyperinsulinemia will result in *β*-cell hyperplasia and hypertrophy [75], which helps to maintain the blood glucose levels up to a point where *β*-cells could no longer produce sufficient insulin to keep pace with the demand [76, 77]. Chronic exposure to high glucose and free fatty acids (FFA) levels could cause progressive *β*-cell dysfunction, which may eventually lead to *β*-cell apoptosis in DM [77, 78]. Research has suggested that insulin secretion from pancreatic *β*-cells is influenced by plasma vitamin D levels [79, 80]. It is proposed that there is a strong correlation between serum vitamin D levels and insulin secretion as well as insulin sensitivity [81, 82]. The role of vitamin D in pancreatic *β*-cell function is supported by the discovery of 1*α*-hydroxylase enzyme, which is classically found in the kidney [83]. Specific staining for 1*α*-hydroxylase was detected in the pancreas and other extra-renal tissues including the colon and brain [83]. Bland et al. reported that 1*α*-hydroxylase is able to convert vitamin D to its active form within the pancreatic islet cells, and this suggests that local production of calcitriol could occur in the pancreatic islet cells [79].

Although the mechanisms underlying the role of vitamin D in pancreatic *β*-cells insulin secretion are not well understood, a few proposals have been made [84]. Kjalarsdottir et al. have shown that *Vdr* mRNA expression in the pancreatic islet cells is glucose responsive [85]. Mice lacking functional VDR are unable to synthesize adequate insulin in response to hyperglycemia [86, 87], and their pancreatic *β*-cells showed a lower amount of stored insulin [87], suggesting vitamin D-dependent insulin synthesis and secretion. In addition to this, a study has demonstrated that VDR expression was reduced when a mouse insulinoma cell line (MIN6) was cultured in a high-glucose environment, and subsequent treatment with vitamin D_3_ improved insulin to the levels seen in normal functional islets in addition to increase in VDR activity as well as prevented the pathological dedifferentiation of *β*-cells [88]. Hence, VDR could be a crucial transcription factor that protects the *β*-cells against dysfunction and maintains its insulin secretion by preventing cell dedifferentiation that precedes *β*-cell failure at the onset of DM. Thus, vitamin D could have a role in maintaining pancreatic *β*-cell function and help to enhance insulin secretion through VDR, and therefore, supplementation with vitamin D could prevent *β*-cell loss and delays the onset of DM. Since insulin secretion is calcium-dependent [89], vitamin D, which is known to be involved in calcium homeostasis, could play an indirect role in pancreatic *β*-cells' insulin secretion. Insulin release requires calcium influx and the opening of voltage-gated calcium channels (VGCCs) upon glucose stimulation [90]. The active form of vitamin D, calcitriol, regulates extracellular calcium levels and calcium influx into *β*-cells via VGCC [91]. It has also been proposed that vitamin D upregulates the *Cacna1e* gene, which encodes the Cav2.3 subunit of R-type VGCC [85]. In a study by Kjalarsdottir et al. on cultured human and mouse pancreatic islets, pre-incubation with vitamin D enhances glucose-stimulated insulin secretion and increases glucose-stimulated calcium influx [85]. Hence, these results suggest that vitamin D could enhance calcium influx through VGCC, which in turn modulates the ability of the pancreatic *β*-cells to secrete insulin [85].

**Figure 1:**
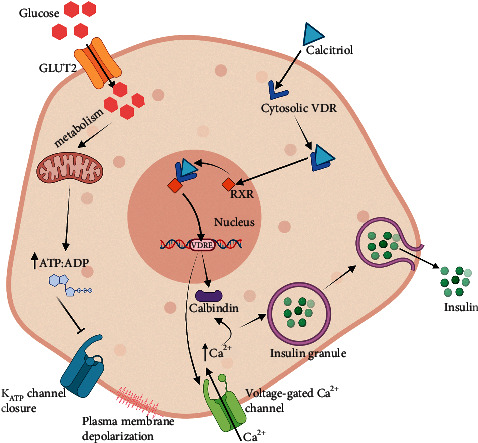
The mechanism underlying glucose-stimulated insulin secretion in pancreatic *β*-cell. Glucose is transported into the cell via GLUT2. Glucose metabolism leads to a high ATP : ADP ratio, which triggers the closure of ATP-sensitive potassium channel (K_ATP_ channel). The resulting plasma membrane depolarization stimulates the opening of the voltage-gated calcium channels (VGCCs) and calcium influx. High intracellular calcium level induces the exocytosis of the insulin secretory granule and insulin secretion. Vitamin D in its active form calcitriol binds to cytosolic VDR. Calcitriol-VDR complexes are translocated into the nucleus and bind to RXR to form calcitriol-VDR-RXR complexes. These complexes then bind to VDRE within the calbindin gene promoter regions to stimulate the transcription of cytosolic calcium-biding proteins, calbindins. calbindins regulate cytosolic calcium concentration and indirectly modulate calcium-dependent insulin secretion. Here, vitamin D also indirectly upregulates the Vgcc genes and thus could help to enhance calcium influx through VGCC.

Although VGCC represents the most common pathway for insulin secretion [92], some studies suggested that calcium-binding proteins or calbindin might have a role in the regulation of intracellular calcium responses in *β*-cells as it is one of the cytosolic vitamin D-dependent calcium-binding proteins found in these cells [93]. Li et al. proposed that calbindin modulates intracellular calcium levels by suppressing calcium-dependent depolarization after-potentials and hence regulates hormonal secretion by acting as an endogenous calcium buffer and controlling depolarization-induced release of a hormone, including insulin [94]. Therefore, vitamin D could indirectly modulate insulin secretion by regulating the levels of calbindin, which controls depolarization-induced insulin release via the regulation of cytosolic calcium concentrations [93]. Although the exact mechanisms of vitamin D regulation of calbindin are not clearly understood, calbindin has been identified as one of the target genes of the *Vdr* [95] and the transcription of vitamin D-dependent calbindin proteins is mediated by the binding of VDR to the functional vitamin D response element (VDRE) within the gene promoter regions [96].  Figure 1 shows the proposed mechanisms underlying vitamin D action in stimulating insulin secretion by the pancreatic *β*-cells.

### 3.2. Vitamin D Improves Insulin Sensitivity

There are several mechanisms by which vitamin D affects insulin sensitivity in insulin target tissues. When vitamin D is deficient, insulin sensitivity will begin to decline, thus setting the stage for the onset of DM and other DM-related illnesses [97]. Firstly, vitamin D modulates the secretion of insulin-sensitizing hormones such as adiponectin and leptin [61] and increases the expression of disulfide-bond A oxidoreductase-like (DsbA-L) protein, a key regulator for adiponectin production [98]. Lower levels of adiponectin have been reported in vitamin D-deficient, obese children [98], whereas higher adiponectin levels were observed in patients with type 2 DM (T2DM) receiving vitamin D-fortified food containing 500 IU vitamin D_3_ daily for twelve (12) weeks [99]. Besides, vitamin D maintains the insulin signaling pathway by increasing the expression of insulin receptors (IRs) [100]. A deficiency in vitamin D could see a decline in IR expression, leading to the onset of insulin resistance [101]. Previous studies found that vitamin D-treated U-937 human promonocytic cells have higher VDR protein expression and *Ir* mRNAs, suggesting that vitamin D is capable of inducing the transcriptional activation of the human *Ir* gene in insulin-responsive cells [101]. Another study has identified the presence of VDR in the human *Ir* gene promoter region in vitamin D-treated U-937 human promonocytic cells, which suggested that vitamin D in the form of calcitriol might enter the insulin-responsive cells and bind to cytosolic VDR prior to the nuclear translocation to further bind to nuclear retinoic acid X-receptor (RXR) in order to form calcitriol-VDR-RXR complex [102, 103]. This complex then binds to the VDRE in the human *Ir* gene promoter region to enhance mRNA expression and transcriptional activation of the *Ir* genes [61]. The increased expression of *Ir* genes could lead to the upregulation of IR, which increases the total number of IR to enhance IR capacity and maintain the insulin sensitivity [101].

Vitamin D has also been found to enhance the insulin action in cells through the PI3K-dependent insulin signaling [103]. The activation of IR will stimulate the phosphorylation of tyrosine residues in the insulin receptor substrate (IRS) protein where its key functions is to regulate the PI3K [104]. Therefore, by modulating the *Ir* gene and upregulating the IR, vitamin D could indirectly boost the IRS-associated PI3K activity, which is involved in the glucose uptake in insulin-responsive tissues [105]. In addition, vitamin D might help to improve the insulin sensitivity by activating peroxisome proliferator-activated receptor delta (PPAR-*δ*), a transcription factor that is involved in the metabolism and mobilization of FFA in the target tissues [61]. A study reported that PPAR-*δ* (NR1C2) knockout mice was found to be glucose intolerant and metabolically less active, while treatment with PPAR-*δ* agonist to diabetic *db*/*db* mice increases insulin sensitivity in all major insulin-responsive tissues [106]. PPAR-*δ* has been reported to ameliorate hyperglycemia by increasing the glucose flux through the pentose phosphate pathway (PPP), thereby enhancing carbohydrate catabolism and suppressing the glucose production in the liver [107]. Additionally, it also helps to increase the *β*-oxidation of FFA in the skeletal muscles, inhibiting FFA release from the adipocytes, and therefore improving the metabolic homeostasis and enhancing the systemic and peripheral insulin sensitivity [107]. Insulin resistance could then trigger lipoprotein lipase to hydrolyze stored TG in inflamed adipose tissue and release the resulting FFA into the circulation, which would then be taken up by other organs such as skeletal muscles and liver, causing excessive fat accumulation and lipotoxicity, which are responsible for the development of insulin resistance [108]. Although the role of vitamin D in activating PPAR-*δ* is unclear, evidences suggested that PPAR-*δ* is the primary vitamin D-responding gene, while PPAR-*δ* and VDR signaling pathways are interconnected by cross-regulation at the level of their transcription factor mRNA [109].

The role of vitamin D in maintaining insulin sensitivity could also be related to its association with VDR and forkhead box protein O1 (FoxO1) protein, where the latter is an important downstream negative regulator in the insulin signaling pathway [110]. Previous study has suggested that FoxO1 regulates IRS-2 protein tyrosine phosphorylation, and thus enhances insulin signal transduction and improves insulin sensitivity [111]. In a study on skeletal muscle-specific VDR-null mice, the mice were found to develop insulin resistance and glucose intolerance with elevated FoxO1 expression and activity [86], which might be responsible for insulin resistance and impaired glucose metabolism in the skeletal muscle [86]. The treatment of C2C12 myoblasts with calcitriol reduced FoxO1 nuclear translocation, expression, and activity, which possibly be VDR-dependent [86]. Thus, vitamin D might play an important role in maintaining peripheral insulin sensitivity through the presence of VDR and indirectly modulating the expression of FoxO1 in insulin-responsive tissues.

**Figure 2:**
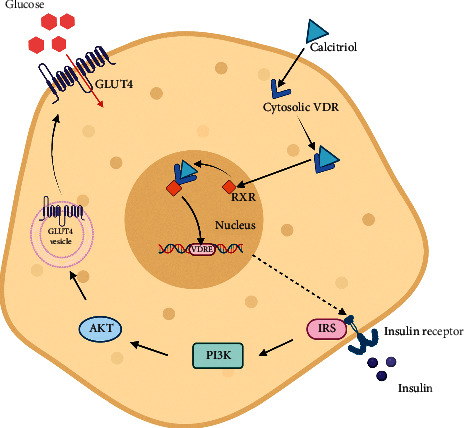
The mechanism underlying insulin-induced glucose uptake in a target cell. vitamin D in its active form, calcitriol binds to cytosolic VDR, which are then translocated into the nucleus and bind to RXR to form calcitriol-VDR-RXR complexes. The complexes then bind to VDRE within the Ir gene promoter regions to stimulate the transcription and upregulation of insulin receptor gene. The binding of insulin to IR stimulates a cascade of process involving multiple downstream mediators, including insulin receptor substrate-1 (IRS-1) and PI3K. The resulting activation of protein kinase B (Akt) stimulates the translocation of glucose transporter type 4 (GLUT4) to the plasma membrane, which facilitates the uptake of circulating glucose into the cell.

Vitamin D could help to upregulate the expression of glucose transporters and its translocation onto the cell membrane, which are essential for the glucose uptake into cells [112]. Evidences have shown that the treatment of L6 myotubes with calcitriol leads to a significant increase in the expression of glucose transporter-1 (GLUT1) and glucose transporter-4 (GLUT4) [113]. Moreover, a study has revealed that vitamin D could help to increase the glucose consumption via inducing SIRT1 activation, which regulates the activation of IRS-1 and GLUT4 translocation in the murine C2C12 myotubes [114]. A study has demonstrated that vitamin D could directly upregulate GLUT4 expression in 3T3L1 adipocyte cell lines and improves the insulin sensitivity [112]. Meanwhile, in the liver, an increase in hepatic insulin signals including phosphorylated Akt (pAkt), phosphorylated FoxO1 (pFoxO1), and phosphorylated glycogen synthase kinase 3 beta (pGSK3*β*), which are involved in glucose transport into the hepatocytes, are observed in C57BL/6 male mice receiving intraperitoneal injections of 50 ng cholecalciferol three (3) times/week for six (6) weeks [19]. Nevertheless, a study performed on C57BL/6J mice showed an increased expression of IRS-1 in the skeletal muscle and increased expression of VDR in the liver, but there was no significant correlation between vitamin D supplementation and GLUT4 expression in other target tissues [115]. In insulin-responsive tissues, a narrow range of intracellular calcium is required for insulin-mediated functions and insulin-associated intracellular processes [116]. High intracellular calcium would enhance GLUT4 translocation onto the cell membrane of the skeletal muscle cells and increases the glucose uptake [117]. Thus, low intracellular calcium in insulin target tissues may impair insulin signal transduction, and this could lead to peripheral insulin resistance [118]. In view of this, vitamin D might affect insulin sensitivity through its role in the regulation of extracellular calcium as well as calcium flux through the cell membranes [93].  Figure 2 shows the proposed mechanisms by which vitamin D acts in order to enhance the glucose uptake in insulin-responsive target tissues.

Apart from these, vitamin D deficiency could also lead to elevated levels of parathyroid hormone (PTH), which has been documented to reduce insulin-stimulated glucose uptake [119]. Although the exact mechanisms are unclear, it is proposed that low serum calcidiol levels could reduce calcium absorption, hence causing secondary hyperparathyroidism [120, 121], which will exacerbate insulin resistance by decreasing the number of GLUT1 and GLUT4 in the adipose tissue, liver, and skeletal muscles [49, 122]. Moreover, PTH treatment in 3T3-L1 adipocytes has been shown to suppress insulin-stimulated glucose uptake and insulin signaling through IRS-1 phosphorylation at serine 307 via cyclic adenosine 3,5-monophosphate (cAMP) pathway [123]. Reduced IRS-1 and GLUT4 expressions will contribute towards lower insulin-induced glucose transport, and these could explain the link between vitamin D deficiency, high serum PTH levels, and insulin resistance [123].

**Figure 3:**
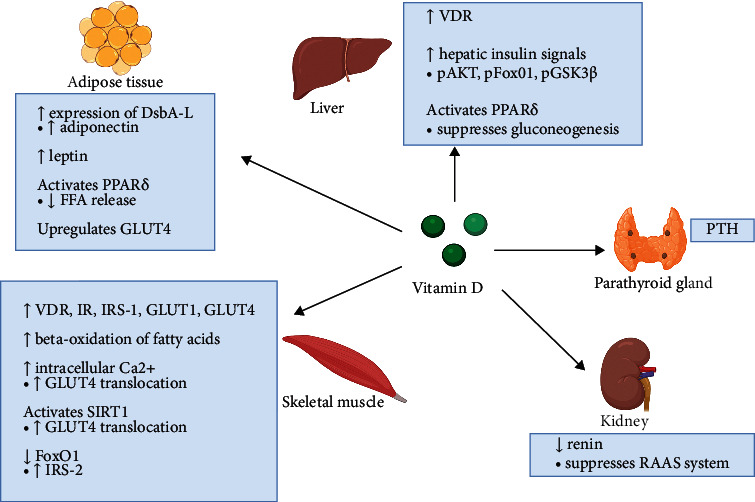
Schematic diagram summarizing the roles of vitamin D in maintaining insulin sensitivity. In the liver, vitamin D increases VDR expression, enhances hepatic insulin signals, and activates PPAR-*δ* to suppress hepatic glucose production. In the adipose tissue, vitamin D increases the production of insulin-sensitizing hormones adiponectin and leptin, activates PPAR-*δ* to reduce the release of FFA into the circulation, and upregulates GLUT4. In the skeletal muscles, vitamin D increases the levels of VDR, IR, IRS-1, GLUT1, and GLUT4, enhances *β*-oxidation of fatty acids, increases intracellular calcium levels, and activates SIRT1 to enhance the translocation of GLUT4 to the plasma membrane for glucose uptake, and increases the level of IRS-1 by suppressing FOXO1. Vitamin D also suppresses the gene expression of renin, thereby preventing inhibitory effects of RAAS against insulin action in peripheral tissues. On the other hand, secondary hyperparathyroidism as a result of vitamin D deficiency could exacerbate insulin resistance by reducing the glucose uptake. Vitamin D could maintain insulin sensitivity by increasing calcium absorption and preventing the secondary elevation of PTH.

In the meantime, vitamin D deficiency could also indirectly affect insulin resistance through the renin-angiotensin-aldosterone system (RAAS). VDR-knockout mice showed higher expressions of renin and angiotensin II, but their levels were reduced following vitamin D administration [124]. Besides, RAAS inhibition could help to improve insulin resistance and glucose intolerance in nondiabetic patients with cardiovascular diseases as well as improve the cardiovascular and renal outcomes in DM [125]. Zhou et al. reported that the inhibition of insulin action in peripheral tissues by RAAS might be mediated via the regulation of intracellular calcium levels where a reduced calcium level will inhibit insulin action [125]. Vitamin D has a genomic effect against RAAS through the suppression of *Renin* gene expression via transcription factor cAMP response element-binding protein (CREB) [126].  Figure 3 summarizes the mechanisms of vitamin D action in various tissues to enhance insulin action and reduces insulin resistance.

### 3.3. Vitamin D Ameliorates Chronic Inflammation

During the development of insulin resistance, chronic low-grade inflammation occurs [127], which can cause impairment in adipose tissue function by causing mitochondrial dysfunction and triggering endoplasmic reticulum (ER) stress—all of which would contribute towards insulin resistance [128, 129]. Although it is unclear whether insulin resistance or inflammatory response occurs first, it was suggested that inflammation in T2DM is the causative factor for insulin resistance [130]. The onset of insulin resistance is believed to occur with the dysregulation of metabolic pathways in the adipose tissue [131]. In obesity-related insulin resistance, poor blood flow in hypertrophied adipose tissue leads to macrophages infiltration due to tissue hypoxia and subsequently inflammation [132]. Adipocyte hypertrophy could result in increased secretion of pro-inflammatory adipokines including tumor necrosis factor *α* (TNF-*α*), interleukins (IL-6, IL-8), monocyte chemoattractant protein 1 (MCP-1), and resistin [133]; and the decrease in the release of anti-inflammatory adipokines such as adiponectin [134]. Studies have shown that vitamin D may act as an anti-inflammatory agent and modulates inflammatory responses by promoting the secretion of anti-inflammatory cytokines and suppressing the secretion of pro-inflammatory cytokines [135, 136].

In a study using diabetic male SD rats, oral supplementation of 0.03 *μ*g/kg/day vitamin D for eight (8) weeks resulted in lower expression of pro-inflammatory cytokines as well as reduced hyperglycemia as reflected by a decrease in FPG levels and HOMA-IR [137]. Moreover, oral supplementation of 150 IU/kg calcitriol per day for sixteen (16) consecutive weeks in high-fat diet (HFD)-induced diabetic C57BL/6J male mice lowered the concentrations of various inflammatory markers including TNF-*α*, C-reactive protein (CRP) and IL-6, and the levels of C-peptide and insulin [138]. These findings were attributed to vitamin D role in modulating inflammatory responses, which attenuated the crosstalk between inflammation and insulin resistance [137]. Apart from this, vitamin D has been reported to suppress the release of several other pro-inflammatory cytokines including IL-6, IL-1, IL-8, COX-2, intercellular adhesion molecule 1 (ICAM-1), and B7-1 protein [139]. Streptozotocin-induced diabetic rats with vitamin D deficiency had enhanced insulin resistance with high proportion of phospho-p65 (p-p65)/RelB in which RelB is an anti-inflammatory molecule, while p-p65 is a pro-inflammatory molecule. An overproduction of pro-inflammatory cytokines not only causes inflammation but also leads to the dysregulation of the glucose and lipid metabolisms [61]. For instance, pro-inflammatory cytokines involved in the activation of I*κ*B kinase beta (IKK-*β*)/nuclear factor kappa B (NF-*κ*B) and c-Jun N-terminal kinase 1 (JNK1) pathways cause serine kinase phosphorylation of IRS-1 or IRS-2, which attenuate insulin signaling and consequently leads to the development of insulin resistance [140].

Studies have demonstrated that vitamin D has an anti-inflammatory role through both NF-*κ*B and p38 MAPK inflammatory pathways [141, 142] with NF-*κ*B being an essential component of the inflammatory pathways in the adipose tissue [143]. The translocation of both NF-*κ*B and p38 MAPK is related to the degradation of inhibitor kappa B-alpha (I*κ*B-*α*) and the transcription of pro-inflammatory genes including IL-6, TNF-*α*, and interleukin 1*β* (IL-1*β*) [144, 145]. Vitamin D upregulates I*κ*B-*α* by reducing I*κ*B-*α* phosphorylation and thereby reduces the nuclear translocation of NF-*κ*B and p38 MAPK, downgrading their pro-inflammatory activities [146].

Evidences also showed that VDR is expressed in immune cells such as the macrophages and dendritic cells [147]. VDR deficiency is linked to inflammation in several diseases, including DM [148]. In fact, vitamin D and VDR have anti-inflammatory and immunosuppressive effects in autoimmunity by increasing the phagocytic ability of monocytes to modulate the innate immune system as well as by promoting the ability of dendritic cells to modulate regulatory T-cell differentiation [149]. In addition, both macrophages and dendritic cells express 25-hydroxylase and 1*α*-hydroxylase enzymes, indicating their role in calcitriol production [150]. Macrophages are also known for cytokine production, and one of the most important inflammatory cytokines secreted by macrophages is TNF-*α* [151]. In dendritic cells, vitamin D elevates the production of anti-inflammatory IL-10 and reduces the release of pro-inflammatory cytokines including TNF-*α*, IL-12, and IFN-*γ* [61]. In addition to this, vitamin D may reduce tissue inflammation by inhibiting the secretion of TNF-*α*-induced chemokine MCP-1 that is responsible for macrophage and monocyte infiltration [58]. In human monocytes, vitamin D suppresses the mRNA expression of Toll-like receptor 2 (*Tlr-2*) and Toll-like receptor 4 (*Tlr-4*) proteins [152], which are important regulators of metabolic inflammation during the development of metabolic disorders [153]. Hence, vitamin D deficiency may exacerbate insulin resistance through an increase in tissue inflammation.

### 3.4. Vitamin D Attenuates Oxidative Stress

Oxidative stress is recognized as a key mechanism in insulin resistance [154]. Among the endogenously produced oxidative stress agents are the reactive oxygen species (ROS), which include superoxide, hydrogen peroxide, and hydroxyl radicals [155]. ROS possesses physiological significance even at low levels, especially to the signaling pathways [156]. The main source for ROS is NADPH oxygenase (NOX) [157] and malondialdehyde (MDA) [158]. The oxidative processes are regulated by antioxidants such as superoxide dismutase (SOD), glutathione (GSH), glutathione peroxidase (GPx), and catalase [159]. Higher production of ROS and declining antioxidative capacity may lead to excessive lipids, proteins, and DNA oxidation products [160]. In oxidative stress, oxidative degradation of lipids causes damage to cell membranes [161], which will eventually lead to cell damage and disruption to the signaling pathways. A study on C2C12 muscle cells showed that vitamin D deficiency could cause mild oxidative stress and an increased muscle proteolysis, while pretreatment with vitamin D could help to reverse oxidative stress and total protein degradation, and reduce muscle atrophy [162]. Besides, vitamin D could help to diminish the ROS formation by downregulating NOX through the suppression of *Nox* gene expression [163, 164]. Studies supported the antioxidant properties of vitamin D where in vitamin D-deficient mice, the inhibition of oxidative stress could improve insulin resistance [165]. In addition to this, a study on SD male weanling rats reported that vitamin D deficiency is linked to a decreased SOD and catalase enzymes in the rat skeletal muscles, and there were higher nitrate levels indicating nitrosative stress in the tissue [162].

Meanwhile, in a randomized double‐blind placebo‐controlled clinical trial on overweight and vitamin D‐deficient women with polycystic ovarian syndrome (PCOS), vitamin D treatment for eight (8) weeks resulted in lower MDA levels and increased production of GSH, which enhances ROS removal [166]. Vitamin D also enhances GSH production by upregulating genes for the key enzymes that are involved in GSH synthesis, such as glutathione reductase (GR), glucose-6-phosphate dehydrogenase (G6PD), and glutamate-cysteine ligase (GCL) [167, 168]. A randomized placebo-controlled trial on women with gestational diabetes mellitus (GDM) reported a significant increase in plasma GSH, lower plasma MDA, and improved metabolic profile with calcium-vitamin D co-supplementation [169]. Another randomized, double-blind, placebo-controlled clinical trial in atopic dermatitis patients confirmed that vitamin D supplementation increases the SOD and catalase activities in erythrocytes [170]. Indeed, a cell culture study using human placental umbilical cord vein endothelial cells reported that oxidative stress downregulates VDR expression in endothelial cells, whereas vitamin D treatment enhances antioxidant enzyme Cu, Zn-superoxide dismutase (SOD1) expression in these cells [171]. A clinical trial on T2DM patients reported a significantly lower vitamin D and GPx levels when compared to healthy individuals; however, there is no statistical correlation between serum vitamin D levels and SOD activity was observed [172].

The mechanisms by which vitamin D alleviates oxidative stress are still a matter of debate. Vitamin D could act by utilizing the genomic mechanisms in ameliorating oxidative stress where an increase in ROS formation could induce the hypermethylation of the gene promoter regions, which could adversely affect the genes that are responsible in the protection against oxidative stress [173] such as the peroxiredoxin 2 (*Prdx2*) gene that encodes a family of antioxidative enzymes and the scavenger receptor class a member 3 (*Scara3*) gene that encodes a scavenger protein that depletes the ROS radicals [174]. By maintaining the expression of DNA demethylases, which reduces the hypermethylation of gene promoter regions, vitamin D could indirectly play a role in reducing ROS levels and provide protection against oxidative stress in the tissue in the insulin resistance state [175].

### 3.5. Vitamin D Abrogates Apoptosis

In the pancreas, unresolved inflammation in the insulin resistance state could enhance the immune cell infiltration, which leads to the dysfunction of insulin-secreting *β*-cells and ultimately cell death [176]. Markedly increased caspase activation and adipocyte apoptosis have been observed in insulin-resistant adipose tissue [177]. In the skeletal muscle, an increase in circulating saturated fatty acids along with poor fatty acid handling results in increased levels of ceramide [178], which acts as a second messenger in triggering an apoptotic response via the mitochondrial system [179]. In the liver, insufficient unfolded protein response (UPR) to elevated ER stress leads to adverse effects such as hepatic fat buildup, inflammation, and cell death [180]. A study on streptozotocin-induced type 1 diabetic FVB mice demonstrated enhanced C/EBP homologous protein (CHOP) and caspase-12 cleavage in response to ER stress in the liver, which resulted in hepatocyte apoptosis [181]. Vitamin D has been reported to be involved in regulating cell proliferation, differentiation, and apoptosis in numerous tissues, including in the insulin-responsive tissues [182]. Firstly, the actions of vitamin D against apoptosis might be attributed to its calcium regulatory role as the molecular targets of vitamin D-mediated apoptosis are calcium-dependent protease calpain and calcium/calpain-dependent caspase-12, which are the primary calcium-activated apoptotic factors [183]. In insulin resistance state, sustained increase in intracellular calcium activates calcium-dependent calpain, which subsequently activates calcium/calpain-dependent caspase-12, resulting in apoptosis [184]. Vitamin D might reduce the intracellular calcium levels, which subsequently prevents calcium-dependent calpain activation and subsequently ameliorating apoptosis.

**Figure 4:**
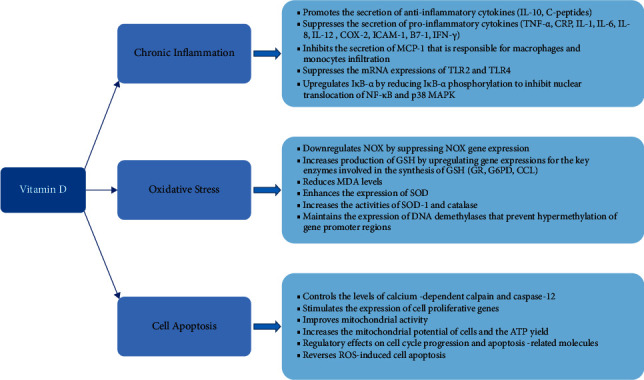
The roles of vitamin D in chronic inflammation, oxidative stress, and cell apoptosis.

Besides, vitamin D could protect the cells against cytokine-induced apoptosis by directly modulating the expression and activity of inflammatory cytokines [139] as well as stimulates the expression of genes that favored cell proliferation in adipocytes [185]. Furthermore, vitamin D may inhibit apoptosis [186] by improving the mitochondrial activity [51] and increasing the mitochondrial potential of cells and adenosine triphosphate (ATP) yield [187], and through the regulatory effects on cell cycle progression and apoptosis-related molecules [52]. These mechanisms may involve the presence of VDR and the regulation of FoxO1. A recent study has demonstrated that VDR gene silencing is associated with reduced cell survival and overexpression of *FoxO1* mRNA and protein, which imply that VDR plays important role in reducing cell death [13]. Vitamin D treatment and high VDR expression have been shown to induce cell survival and mitigate FoxO1-induced cell apoptosis, whereas vitamin D treatment and FoxO1 gene silencing reverse ROS-induced cell apoptosis [13].  Figure 4 shows the roles of vitamin D in ameliorating inflammation, oxidative stress, and apoptosis in tissues.

## 4. Conclusion

Vitamin D seems to have a significant role in metabolism, and its deficiency could be linked to the pathogenesis of insulin resistance. Insufficient levels of vitamin D are associated with hyperglycemia, low insulin sensitivity, chronic inflammation, oxidative stress, and apoptosis. Normal vitamin D levels are associated with normal glucose homeostasis, insulin sensitivity, improved pancreatic *β*-cell function and insulin secretion, and other improvement in metabolic parameters. Therefore, prompt detection and effective management of vitamin D deficiency in individuals with insulin resistance could be an easier, cost-effective approach that may improve the health outcomes and help to reduce the risk of developing DM and other related metabolic disorders.

## Figures and Tables

**Table 1:** Animal experimental studies on the effects of vitamin D on insulin resistance.

Model	Treatment	Findings	References
HFD-induced C57BL/6J male mice	Oral 150 IU/kg per day calcitriol orally (in 1 mL coconut oil) by oral gavage for 16 weeks	Gradual decrease in weight in HFD-fed mice treated with calcitriol, reduced concentrations of various inflammatory markers including TNF-*α*, CRP, and IL-6, lower the levels of C-peptide and insulin, improved liver structure	Alkharfy et al. [115]

Diet-induced obese C57BL/6J WT mice	12 weeks of 60% HFD with 9–12 weeks of calcipotriol (TOCRIS) treatment (20 *μ*g/kg body weight)	VDR activation by calcipotriol suppressed liver inflammation and hepatic steatosis, therefore significantly improving insulin sensitivity	Dong et al. [59]

Vitamin D-deficient C57BL/6 male mice	Intraperitoneal injections of cholecalciferol 50 ng·3x/week for 6 weeks	Improved insulin sensitivity in vitamin D-deficient lean mice but no significant improvement in insulin resistance in obese mice	Mutt et al. [19]

Diet-induced obese C57BL/6J male mice	Intraperitoneal injections of 7 *μ*g/kg calcitriol 3x/week for 8 weeks	Reduced body weight, improved overall systemic glucose tolerance, restored insulin signaling, and reverted hepatic myosteatosis	Benetti et al. [57]

Diet-induced obese and insulin-resistant C57BL/6J adult male mice	Oral calcitriol 3000 IU/kg/day (75 mg/kg/day) for 7 consecutive days	Complete restoration of insulin sensitivity, reduced body weight, and glycemia, but with severe kidney damage	Gaspar et al. [55]

Diet-induced vitamin D-deficient obese C57BL/6J male mice	Oral cholecalciferol 67 IU/kg/day for 8 weeks	Upregulated glucose uptake, improved glucose metabolism, prevented oxidative stress via novel molecular mechanisms	Manna et al. [54]

Diet- and STZ-induced diabetic male SD rats	Oral vitamin D 0.03 *μ*g/kg/day for 8 weeks	Protective effects against diabetes-induced liver complications by attenuating the crosstalk between inflammation and insulin resistance, and ameliorating hyperglycemic state	Liu et al. [137]

STZ-induced diabetic male SD rats	Intraperitoneal injections of 20,000 IU/kg of cholecalciferol on days 1 and 14	Significant decrease in fasting plasma glucose, decline in HbA1c, improved insulin, and IGF-1 levels	Derakhshanian et al. [56]

Alloxan-induced diabetic female albino mice	7 ng/gm/day of 1,25(OH)2D3 dissolved in propylene glycol given intraperitoneally for 15 days	Lowered serum glucose, improved activities of enzymes of glucose metabolic pathways, restored glucose homeostasis, and reduced pancreatic and liver damage.	Meerza et al. [53]

**Table 2:** Interventional clinical trials on the effects of vitamin D supplementation on insulin sensitivity.

Study design	Population of study	Intervention	Findings	References
Double-blind, placebo-controlled, randomized clinical trial	44 participants with serum 25(OH)D level ≤50 nmol/L and BMI 30–40 kg/m^2^	Weight reduction diet with 50,000 IU vitamin D_3_ pearl once a week for 12 weeks	Improved fasting serum glucose and matrix metalloproteinase 9 (MMP-9) levels; no significant differences for glycemic markers (serum insulin, HOMA-IR)	Aliashrafi et al. [66]

Single-blinded randomized control trial	100 diabetic pregnant women	60,000 IU of oral vitamin D_3_ once a month till delivery	No improvement in insulin resistance or glycemic control in diabetic pregnant women with vitamin D deficiency	Bhavya Swetha et al. [69]

Double-blind, placebo-controlled clinical trial	160 post-menopausal women aged 50–65 years old	Daily 1000 IU of oral vitamin D_3_ for 9 months	Reduction in metabolic syndrome risk profile in younger post-menopausal women with vitamin D deficiency	Ferreira et al. [81]

Single-center, double-blind, randomized placebo-controlled trial	150 healthy premenopausal women with vitamin D insufficiency	20,000 IU of oral cholecalciferol weekly for 24 weeks	Significant improvement in HOMA-IR and QUICKI, no significant effect on AUCgluc.	Trummer et al. [68]

Double-blind randomized clinical trial	90 obese type 2 diabetes patients	50,000 IU of oral vitamin D pearls weekly for 8 weeks	Significant decrease in HbA1c and improved T2D but no significant changes in glucose indices (FPG, insulin, HOMA-IR, QUICKI)	Safarpour et al. [65]

Double-blind, randomized, placebo-controlled trial	18 obese, nondiabetic, vitamin D-deficient volunteers	25,000 IU oral cholecalciferol weekly For 3 months and lifestyle modification	Improved insulin sensitivity and body composition but no improvements in pancreatic *β*-cell function	Cefalo et al. [62]

Double-blind randomized clinical trial	162 prediabetic, vitamin D-deficient subjects	50,000 IU of oral vitamin D_3_ pearls weekly for 3 months, followed by 1 pearl per month	Improved insulin sensitivity and decreased rate of progression toward overt diabetes	Niroomand et al. [63]

Randomized controlled trial	92 vitamin-D-deficient subjects	Daily 2000 IU oral cholecalciferol for 3 months	Decreased level of DNA damage, reduced insulin resistance parameters, and improved glucose and lipid metabolisms	Wenclewska et al. [64]

Randomized, placebo-controlled, double-blinded trial	50 female subjects (20 to 40 years old) with PCOS and vitamin D deficiency	50,000 IU of oral vitamin D3 or placebo, once every 20 days for 2 months	There were no significant changes in fasting serum insulin and glucose levels, and HOMA-IR	Ardabili et al. [70]

## Data Availability

The datasets supporting the conclusions of this study are included within the article.
